# Validation of a Self-reported Physical Activity Questionnaire for Schoolchildren

**DOI:** 10.2188/jea.13.278

**Published:** 2007-11-30

**Authors:** Xiaoli Chen, Michikazu Sekine, Shimako Hamanishi, Hongbing Wang, Alexandru Gaina, Takashi Yamagami, Sadanobu Kagamimori

**Affiliations:** 1 Department of Welfare Promotion and Epidemiology, Faculty of Medicine, Toyama Medical and Pharmaceutical University.; 2 Hokuriku Health Service Association.

**Keywords:** validity, motor activity, energy metabolism, observational studies

## Abstract

BACKGROUND: There is little information about validation of young children’s self-reported physical activity. This study assessed the validity of a self-reported questionnaire designed to measure children’s physical activity.

METHODS: Subjects were 34 boys from 4th to 6th grade of a public elementary school. Contents of the self-reported physical activity questionnaire included participation in sports club, physical activity intensity, preference for physical activity, and frequency of physical activity. Subjects were equipped with a Lifecorder and an Actiwatch for 7 consecutive days to monitor physical activity. Physical activity index was calculated from the Lifecorder data of total energy expenditure per day (TEE) divided by basal metabolic rate. Unpaired t-test, analysis of variance, and multiple linear regression analysis were performed to clarify the relationship between the objective and subjective data.

RESULTS: Subjects who reported participation in a sports club had a higher physical activity index and energy expenditure originating from physical activity than those who did not. Those characterized by a “vigorous” physical activity intensity had a higher physical activity index (1.63±0.08), when compared with peers in “moderate” (1.59±0.06) or “light” categories (1.54±0.07) (p for linear trend p<0.05). A high frequency of physical activity was significantly associated with an increasing trend in energy expenditure originating from physical activity, steps, and activity counts. Preference for physical activity was significantly related to data from the Lifecorder and the Actiwatch.

CONCLUSIONS: Schoolchildren’s self-reported physical activity is in accordance with the objective data, and could be used as a valid measure to evaluate physical activity level in school settings.

Methods of assessing physical activity in child populations have included direct observation, electronic motion sensors and heart rate, double-labeled water, and self-report.^1^^-^^5^ Although direct observation is a useful measure for methodological studies, it is not practical for a larger population, especially in epidemiologic studies.^1^^,^^6^ Primarily due to considerations of expense and convenience, self-reports are the most commonly used in large field studies.^4^^,^^7^ So far, four types of self-report measures have been categorized as interviewer administered, self-administered, diary, and proxy report (parents or teachers).^4^^,^^8^^,^^9^ There are concerns about the reliability and validity of these measures, although there is growing evidence that children as young as about 10 years of age can provide self-reports of at least modest reliability and validity.^4^^,^^7^ The capacity of children and adolescents to accurately report physical activity is age related and 9-year-old children do not perform adequately.^7^

The purpose of this study was to examine the validity of physical activity self-reports for schoolchildren from 4th to 6th grades. The study was undertaken at an elementary school, in order to establish whether children’s subjective reports on physical activity could be validated by the use of two small instruments, namely the Actiwatch and Lifecorder activity monitors, as criterion measures. Because of the limitations of all objective criteria commonly used to validate physical activity self-reports,^4^^,^^10^ both the Actiwatch and the Lifecorder were used as validation criteria.

## METHODS

### Subjects

Subjects were schoolchildren from 4th to 6th grade of a public elementary school located in Toyama city, Japan. Of all 67 boys in these three grades, 44 boys agreed to participate in the study. Permission in the form of signed consent was obtained from their parents. These boys were in good health or were free of current health problems when they were enrolled into this study. During the observation periods, two of the 44 subjects reported an allergy resulting from wearing the instruments, and consequently the observations on them stopped. Two boys’ data were eliminated from the analysis due to obesity. Data from one boy was unavailable due Actiwatch malfunction and four boys did not have sufficient Actiwatch or Lifecorder data during the seven consecutive days. One suffered a sudden asthma attack during the measurement period and this data was excluded. Therefore, the subjects in the present study consist of 34 boys (mean age: 10.8 years, standard deviation: 0.8 year).

### Self-administered Questionnaire

Contents of the self-administered questionnaire on children’s physical activity and outdoors playing used in this study included: participation in any sports club after school, rated on a two-point scale (1:“yes”, and 2:“no”); frequency of participating in physical activity or outdoors playing during one week, rated on a three-point scale (1:“very often”, 2:“fairly often”, and 3:“not often”); physical activity intensity, rated on a three-point scale: 1:“vigorous” (for example: run, fast pace), 2:“moderate” (for example, walk, medium pace), and 3:“light” (for example, walk, carry light load); and preference for physical activity or outdoors playing, rated on a three-point scale (1:“like very much”, 2:“like”, and 3:“dislike”). Children were also required to complete an additional section to report on their habits of television watching and video games playing.

### Actiwatch and Lifecorder:

The Actiwatch (Mini Mitter Company, Inc., Bend, OR, USA) is a small, lightweight (17 g), limb-worn, activity-monitoring watch-like device, which was designed for the long time monitoring of gross motor activity in human subjects. To date, the Actiwatch has been used in many clinical research studies, mainly for describing sleep and physical activity.^11^^-^^14^ The accelerometer-based activity monitor has proved to be a valid and useful device for the assessment of children’s physical activity, defined as body movement produced by the skeletal muscles resulting in energy expenditure.^13^ Puyau reported that the validation of the CSA and Mini-Mitter Actiwatch monitors against activity energy expenditure, and their calibration for sedentary, light, moderate, and vigorous thresholds certify these monitors as valid, useful devices for the assessment of physical activity in children.^13^

The Actiwatch activity monitor contains an omni-directional sensor capable of detecting acceleration in two planes. Sensitive to 0.01 gravity (0.098 m/s^2^), this type of sensor integrates the degree and speed of motion and produces an electrical current that varies in magnitude. An increased degree of speed and motion produces an increase in voltage. The monitor stores these data as activity counts. This device measures long-term gross motor activity and ambient light exposure.^15^ Activity counts are calculated based on the sampling epoch and the total number of activity counts is compared to the threshold sensitivity value selected by the researcher. In this study, a 1-minute sampling epoch was used and the threshold sensitivity “Auto” was selected. Mean activity is the average activity counts during the 24 hours; Diurnal activity is the magnitude of activity each day during the diurnal time period from 7:30 am through 8 pm; Nocturnal activity is the daily magnitude of nighttime activity from 8 pm through 7:30 am. The time spent immobile and activity score during sleep periods can also be measured.

The Lifecorder (Kenz Activity Monitor, Suzuken CO., Ltd., Japan) is a small instrument with the dimensions of 62.5mm × 46.5mm × 26.0mm and a weight of 40 g including a battery. It can calculate daily steps, energy expenditure originating from physical activity, and total energy expenditure per day, as well as a subject’s basal metabolic rate, physical activity intensity (for example, jogging, fast-walking, or slow-walking), and the maximum physical activity in one day during the measurement periods. The instrument has been used in several aspects of research and confirmed to be a valid measure for estimating physical activity level, although it was designed originally for adult use.^16^^-^^18^ Detailed information on the methodology used for calculating the indices obtained from this small instrument has been published.^18^^,^^19^ The Lifecorder method has merit, in that the measurement of daily activity patterns and daily variation in energy expenditure is similar to that of the Flex HR methods.^18^

The Lifecorder contains a small counter measuring amplitude and frequency of acceleration of body movement every 4 seconds and grades the intensity as one of 10 levels. The graded intensity is transformed into the corresponding coefficient of physical activity (Ka; coefficient as determined by the level of physical activity (kcal/kg/4 seconds). The energy expenditure originating from physical activity per 4 seconds(C) is calculated as Ka × W (weight in kg).

According to recommended dietary allowances published by the Ministry of Health and Welfare (1994),^20^ the basal metabolic rate (BMR) is calculated as follows:

BMR = Kb×A×T,

where ‘Kb’ represents the standard value for basal metabolism per body surface area (kcal/m^2^/hr), ‘A’ represents body surface area(m^2^), and ‘T’ is the time of 24 hours. The equation of body surface area (A) for 6 year olds and over, is calculated as follows: A (cm^2^) = W (kg)^0.444^ × H (cm)^0.663^ × 88.83. Because subjects in this study were boys aged 10-12 years, we used the above equation to obtain children’s BMR.

Energy expenditure originating from minimum body movement (X [kcal/4sec]) is calculated every 4 seconds as follows: X = Kx × B (Kx: coefficient for minimum body movement as estimated by energy metabolic rate; B: basal metabolism). Finally the total energy expenditure (E) is calculated as follows: E= 10/9(B+C+X). The total energy expenditure and energy expenditure originating from physical activity are calculated every 4 seconds and summed over 24 hours (total energy expenditure per day, energy expenditure originating from physical activity). The physical activity index is calculated as follows: physical activity index = total energy expenditure per day / BMR (BMR: basal metabolic rate). Daily steps are automatically counted by the Lifecorder. This can be used to make an assessment of Lifecorder lifestyle activities, so that consultation and prescribing can be based on reports from the Lifecorder data.

### Procedures

The survey was conducted from July through September in 2002, as a sub-study of the Toyama Birth Cohort Study in Toyama Prefecture, Japan. Anthropometric measurements were conducted in the school nursery room. The heights and weights of children in their underwear were measured twice by one trained examiner. Height was measured to the nearest 0.1 cm by using a rigid stadiometer, while weight was measured to the nearest 0.1 kg by using a rigid weighing scale. The Actiwatch was attached to the subject’s non-dominant wrist and the Lifecorder was also attached simultaneously to the waist by means of a belt. After investigators had recorded the attachment time of the Lifecorder, the monitoring of the children’s physical activity for seven consecutive days (including weekdays and weekends) started. Since the Lifecorder is not waterproof, it was removed when the children took a bath or shower. The two monitoring instruments were also detached in the school nursery room by the investigators, when the detachment time was recorded and the self-report on physical activity was checked for completeness. At the same time, investigators also checked data originating from the Actiwatch and the Lifecorder together with the children, in order to confirm the accuracy of possible events during the measurement period.

In relation to the nocturnal and daytime activity monitoring from the Actiwatch, daytime recording was determined from 7:30 am and nocturnal recording was from 8:00 pm.

### Statistical analysis

The difference in objective activity parameters between subjects who reported participating in sports club and those who did not was evaluated by the unpaired t-test. A test for linear trend in analysis of variance (ANOVA) was performed to evaluate the significance of the linear trend of the mean of the objective parameters among the groups with different self-reported physical activity scales. Furthermore, we used the Scheffe’s test of multiple comparison to assess the difference between the different physical activity scales, if the p value was less than 0.05 by ANOVA. Multiple linear regression analysis was performed to clarify the linear trend in the relationship between the objective data and self-reported physical activity level, adjusted for children’s age and body mass index (BMI).

All statistical analyses were performed by the SPSS^®^ 10.0J software package. A two-tailed p value of less than 0.05 was considered to be significant.

## RESULTS

**Figure 1.**
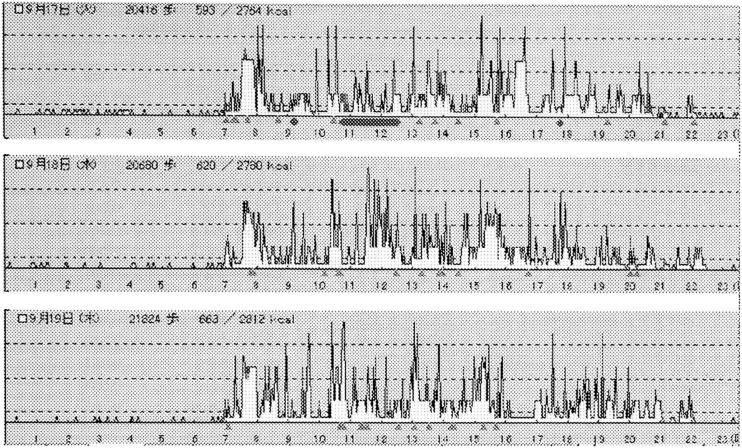
Lifecorder data over three consecutive days for a 10-year-old boy. The boy rated himself with “vigorous” physical activity, a preference for physical activity “like very much”. Daily steps were 23601 steps and the physical activity energy expenditure was 352.9kcal.

**Figure 2.**
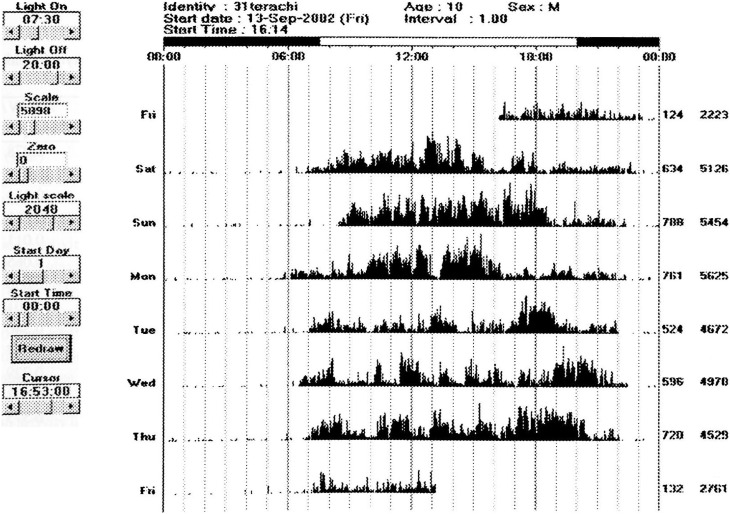
Actigraphic data over seven consecutive days for a 10-year-old boy. The boy rated himself with “vigorous” physical activity, a preference for physical activity “like very much”. The mean activity was 670.5 counts. The highest number of activity counts for a single sampling epoch was 5625 during the study period. Activity: leg activity data compressed to one-minute epoch. Diurnal time: 7:30 am through 8 pm; nocturnal time: 8 pm through 7:30 am.

Figures 1 and 2 show representative Lifecorder and Actigraphic data obtained from one boy aged 10 years old. The boy who rated himself as participating in a sports club after school, with “vigorous” physical activity intensity, a preference for physical activity “like very much”, and a frequency of physical activity practices “quite often”, attained an average step count of 23601 steps per day and expended an average activity energy of 352.9 kcal/day during the 7 consecutive days (Figure 1). According to data from the Actiwatch, the boy had an average activity count of 670.5 per day (minimum: 524; maximum: 788) and the highest activity count of 5625 during the 7 consecutive days (figure 2).

Table 1 presents the results of a comparison between children who participated in sports club with those who did not. Subjects who reported participating in sports clubs after school had a significantly higher physical activity index (1.62 ± 0.07 [mean ± standard deviation) than those who did not (1.54 ± 0.05, p<0.01). Concerning the variables of energy expenditure originating from physical activity, steps, and maximum energy expenditure originating from physical activity and steps, these were significantly higher in children who participated in sports clubs than in their peers who did not (p for a linear trend: p<0.01, p<0.001, p<0.05, and p<0.01,respectively). Data originating from the Actiwatch showed that differences in mean activity score during sleep periods were not found between children who participated in sports clubs and those who did not. There were no significant differences between the two groups in age, height, weight, and BMI.

**Table 1.** Distribution of variables originating from Actiwatch and Lifecorder comparing activity variables of children who participated in sports club with those who did not.

	participate in sports club						t test p value

	Yes (n=24)			No (n=10)			

	Mean	±	SD	Mean	±	SD	
age (year)	10.8	±	0.9	10.9	±	0.7	0.635
weight (kg)	34.4	±	5.9	36.8	±	9.3	0.460
height (cm)	140.8	±	7.5	141.4	±	5.9	0.831
body mass index (kg/m^2^)	17.2	±	1.9	18.2	±	3.5	0.425
physical activity indes	1.62	±	0.07	1.54	±	0.05	0.004
TEE (kcal/day)	2002.1	±	236.8	2101.6	±	394.3	0.369
EEPA (kcal/day)	288.9	±	61.1	212.4	±	49.4	0.002
steps (step/day)	17925	±	2983	13206	±	3649	<0.001
maximum EEPA(kcal/day)	466.9	±	116.6	375.3	±	116.0	0.045
maximum steps (step/day)	24267	±	5588	18074	±	4659	0.004
immobile time (%)	84.1	±	3.5	85.5	±	1.9	0.242
mean activity score (count/day)	21.2	±	14.5	17.6	±	5.9	0.461
mean activity (count/day)	478.9	±	85.4	423.6	±	74.6	0.084
diurnal activity (count/day)	782.7	±	163.4	684.5	±	140.9	0.107

SD: standard deviation

TEE: total energy expenditure per day

EEPA: energy expenditure originating from physical activity per day

immobile time (%): amount of assumed sleep time that is spent immobile

mean activity score: the magnitude of activity on a per-epoch-basis during the sleep period

Table 2 shows the results of testing for a linear trend and Scheffe’s test for multiple comparison of the mean of the variables among subjects of different physical activity intensity. Children who rated themselves as “vigorous” had a mean physical activity index of 1.63 ± 0.08, those rating themselves of “moderate” physical activity intensity had a mean physical activity index of 1.59 ± 0.06, and those of “light” physical activity intensity had a mean physical activity index of 1.54 ± 0.07 (p for a linear trend test: p<0.05). In respect of mean steps per day and maximum steps per day during the 7 days, there were significant differences between the same three groups of children (p for a linear trend test: p=0.001 and p<0.05, respectively). The Scheffe’s test shows that a significant difference existed between subjects of “vigorous” or “light” physical activity intensity in physical activity index (p<0.05), mean steps per day (p<0.01), and maximum steps per day (p<0.05). Data originating from the Actiwatch shows that differences in mean activity score during sleep periods were not found between children of different physical activity intensity as evaluated by the children. Although an increasing trend was also observed in age, height, weight, and BMI, this trend was not significant except for weight.

**Table 2.** Children’s physical activity intensity evaluated by self-administered questionnaire and the activity variables originating from Actiwatch and Lifecorder.

	vigorous (n=12)			moderate (n=15)			light (n=7)			ANOVA

	mean	±	SD	mean	±	SD	mean	±	SD	Trend p value
age (year)	10.5	±	0.9	10.9	±	0.8	11.2	±	0.4	0.078
weight (kg)	32.4	±	5.8	35.3	±	5.3	39.2	±	10.5	0.041
height (cm)	139.1	±	8.0	141.7	±	6.3	142.7	±	6.7	0.251
body mass index (kg/m^2^)	16.7	±	2.2	17.5	±	1.5	18.9	±	3.8	0.052
physical activity index	1.63	±	0.08*^a^	1.59	±	0.06	1.54	±	0.07	0.011
TEE (kcal/day)	1959.9	±	237.0	1994.2	±	209.9	2233.6	±	439.0	0.065
EEPA (kcal/day)	289.0	±	61.2	272.5	±	64.5	214.8	±	65.6	0.036
steps (step/day)	18478	±	3775**^a^, *^b^	16765	±	2633	12720	±	3650	0.001
Max EEPA (kcal/day)	455.3	±	137.2	449.1	±	97.5	394.0	±	149.6	0.340
Max steps (step/day)	24425	±	7317*^a^	23263	±	3996	17299	±	4640	0.017
immobile time (%)	84.9	±	2.8	84.2	±	3.8	84.5	±	2.4	0.729
mean activity score (count/day)	18.1	±	7.5	21.7	±	17.9	20.3	±	4.4	0.643
mean activity (count/day)	469.9	±	83.9	464.8	±	91.9	445.4	±	82.5	0.575
diurnal activity (count/day)	778.0	±	151.1	756.7	±	179.2	706.2	±	151.2	0.375

SD: standard deviation

TEE: total energy expenditure per day

EEPA: energy expenditure originating from physical activity per day

immobile time (%): amount of assumed sleep time that is spent immobile

mean activity score: the magnitude of activity on a per-epoch-basis during the sleep period

*^a^ vigorous vs light, p<0.05; **^a^ vigorous vs light, p<0.01;

*^b^ vigorous vs moderate, p<0.05

Table 3 shows the results of the linear trend test and Scheffe’s tests for the relationship between objective variables from the two monitoring instruments and the frequency of physical activity reported by the children. Subjects with a high frequency of taking physical activity were significantly associated with an increasing trend in energy expenditure originating from physical activity, mean steps, maximum energy expenditure originating from physical activity, maximum steps, and mean activity counts per day originating from the Actiwatch (p for a linear trend test: p<0.05, p=0.001, p<0.01, p<0.001, and p<0.05, respectively). The Scheffe’s test shows that significant differences existed between the following subjects: for those who took physical activity “very often” or “fairly often”, in mean steps per day (p<0.05), maximum energy expenditure originating from physical activity (p<0.05), and maximum steps (p=0.001); for subjects taking physical activity “fairly often” or “not often”, in mean steps per day (p<0.05) and maximum steps (p<0.01). Data originating from the Actiwatch shows that differences in mean activity score during sleep periods were not found between subjects with a different frequency of taking physical activity. There were no significant trends for height and BMI. However, an increasing trend was observed in age (p<0.01) and weight (p<0.05).

**Table 3.** Frequency of physical activity and the activity variable originating from the Lifecorder and Actiwatch.

	very often (n=16)			fairly often (n=14)			not often (n=14)			ANOVA

	mean	±	SD	mean	±	SD	mean	±	SD	trend p value
age (year)	10.4	±	0.8**^a^	11.0	±	0.6	11.8	±	0.3	0.001
weight (kg)	33.2	±	6.2	35.3	±	7.4	42.0	±	4.6	0.035
height (cm)	139.3	±	8.3	141.6	±	5.3	145.4	±	4.3	0.108
body mass index (kg/m^2^)	16.9	±	2.0	17.4	±	2.7	19.8	±	2.2	0.063
physical activity index	1.62	±	0.06	1.58	±	0.08	1.58	±	0.08	0.214
TEE (kcal/day)	1965.1	±	239.4	2075.5	±	356.5	2141.9	±	184.1	0.195
EEPA (kcal/day)	299.0	±	63.6	240.5	±	54.4	233.5	±	76.1	0.014
steps (step/day)	18594	±	3194*^a^, *^b^	15298	±	3353	12645	±	3303	0.001
maximum EEPA (kcal/day)	506.4	±	98.0*^b^	380.7	±	107.2	381.8	±	148.9	0.006
maximum steps (step/day)	26561	±	4511**^a^, **^b^	19342	±	4605	16844	±	4892	<0.001
immobile time (%)	84.8	±	3.3	84.4	±	3.3	84.0	±	2.4	0.645
mean activity score (count/day)	18.0	±	5.4	22.7	±	19.0	19.8	±	1.8	0.513
mean activity (count/d)	500.9	±	92.3	433.1	±	59.4	413.0	±	82.3	0.014
diurnal activity (count/d)	828.3	±	175.3	698.7	±	117.1	648.8	±	124.5	0.010

SD: standard deviation

TEE: total energy expenditure per day

EEPA: energy expenditure originating from physical activity per day

immobile time (%): amount of assumed sleep time that is spent immobile

mean activity score: the magnitude of activity on a per-epoch-basis during the sleep period

*^a^ very often vs not often, p<0.05; **^a^ very often vs not often, p<0.01;

*^b^ very often vs fairly often, p<0.05; **^b^ very often vs fairly often, p<0.01

Table 4 shows the results of the linear trend test and the Scheffe’s tests of the mean of the variables among groups with different physical activity preferences. Subjects with a preference for physical activity were significantly associated with an increasing trend in energy expenditure originating from physical activity, mean steps, maximum energy expenditure originating from physical activity, and maximum steps (p for a linear trend: p<0.05, p=0.001, p<0.05, and p<0.001, respectively). The Scheffe’s test shows that significant differences existed between the following subjects: those who rated themselves as “like very much” or “like”, in energy expenditure originating from physical activity (p<0.05), mean steps per day (p<0.01), maximum energy expenditure originating from physical activity (p<0.05), maximum steps (p<0.001), those subjects who rated themselves as “like very much” or “dislike” in mean steps per day (p<0.05) and maximum steps (p<0.05). Data originating from the Actiwatch shows that differences in mean activity score during the sleep periods were not found between subjects with a different frequency of physical activity. There were no significant trends for height and BMI. However, an increasing trend was observed for age and weight.

**Table 4.** Preferences for self-reported physical activity and the activity variables originating from the Lifecorder and the Actiwatch.

	like very much (n=24)			like (n=7)			do not like (n=3)			ANOVA

	mean	±	SD	mean	±	SD	mean	±	SD	trend p value
age (year)	10.6	±	0.7*^a^	11.3	±	0.8	11.8	±	0.3	0.003
weight (kg)	33.7	±	5.9	36.8	±	9.4	42.1	±	5.7	0.036
height (cm)	139.8	±	6.8	142.0	±	7.5	147.5	±	1.4	0.076
body mass index (kg/m^2^)	17.1	±	2.1	18.0	±	3.4	19.3	±	2.3	0.108
physical activity indes	1.61	±	0.06	1.56	±	0.12	1.59	±	0.09	0.383
TEE (kcal/day)	1963.7	±	210.5	2198.1	±	462.9	2184.0	±	200.6	0.053
EEPA (kcal/day)	288.7	±	58.3**^b^	195.3	±	38.2	248.2	±	86.1	0.022
steps (step/day)	17966	±	3070**^b^	13046	±	3415	13252	±	3762	0.001
maximum EEPA (kcal/day)	473.3	±	100.3*^b^	339.9	±	131.0	407.0	±	171.5	0.048
maximum steps (step/day)	25110	±	4587*^a^,**^b^	15318	±	2789	17760	±	5554	<0.001
immobile time (%)	84.7	±	3.3	84.5	±	3.3	83.4	±	2.6	0.569
mean activity score (count/day)	19.8	±	14.5	21.0	±	8.9	20.5	±	1.5	0.856
mean activity (count/day)	480.7	±	84.2	425.8	±	70.1	401.0	±	80.6	0.035
diurnal activity (count/day)	787.0	±	163.8	692.7	±	131.2	628.0	±	119.2	0.033

SD: standard deviation

TEE: total energy expenditure per day

EEPA: energy expenditure originating from physical activity per day

immobile time (%): amount of assumed sleep time that is spent immobile

mean activity score: the magnitude of activity on a per-epoch-basis during the sleep period

*^a^ like very much vs do not like, p<0.05; **^a^ like very much vs do not like, p<0.01;

*^b^ like very much vs like, p<0.05; **^b^ like very much vs like, p<0.01

To control for potential confounding factors such as age and BMI, multiple linear regression analysis was performed, with the objective indicators from the two instruments as dependent variables; age, BMI, and the self-reported physical activity levels being the independent variables. Each category of self-reported questionnaire items was treated as a continuous variable in the regression models. Table 5 summarizes the results from multiple linear regression analysis. It shows that, after adjusting for children’s age and BMI, the association of objective indicators from the two instruments with self-reported physical activity levels remained significant in most regression models.

**Table 5.** Multiple linear regression analysis for associations between the objective data and self-reported questionnaire items.

	R^2^	age *β*	BMI *β*	questionnaire *β*
sports club				
physical activity index	0.26	0.12	-0.01	-0.51**
TEE (kcal/day)	0.65	0.24*	0.72***	-0.17
EEPA (kcal/day)	0.34	-0.24	0.23	-0.56***
steps (step/day)	0.56	-0.25	0.33*	-0.49***
maximum EEPA (kcal/day)	0.26	-0.40*	0.17	-0.35*
maximum steps (step/day)	0.60	-0.55***	-0.12	-0.41**
mean activity (count/day)	0.24	-0.32	-0.12	-0.25

physical activity intensity				
physical activity index	0.25	0.23	0.05	-0.54**
TEE (kcal/day)	0.63	0.27*	0.73***	-0.15
EEPA (kcal/day)	0.19	-0.16	0.23	-0.42*
steps (step/day)	0.44	-0.19	-0.33*	-0.37*
maximum EEPA (kcal/day)	0.16	-0.38*	0.14	-0.10
maximum steps (step/day)	0.47	-0.53**	-0.14	-0.20
mean activity (count/day)	0.18	-0.34	-0.18	0.07

frequency of physical activity				
physical activity index	0.10	0.28	-0.08	-0.35*
TEE (kcal/day)	0.65	0.35*	0.72*	-0.23^†^
EEPA (kcal/day)	0.21	-0.03	0.17	-0.47*
steps (step/day)	0.44	-0.07	-0.36*	-0.40*
maximum EEPA (kcal/day)	0.27	-0.21	0.18	-0.41*
maximum steps (step/day)	0.55	-0.37*	-0.13	-0.40*
mean activity (count/day)	0.23	-0.20	-0.13	-0.27

preference for physical activity				
physical activity index	0.06	0.22	-0.11	-0.24
TEE (kcal/day)	0.64	0.31*	0.70***	-0.16
EEPA (kcal/day)	0.18	-0.06	0.16	-0.42*
steps (step/day)	0.44	-0.09	-0.37*	-0.39*
maximum EEPA (kcal/day)	0.19	-0.30	0.15	-0.23
maximum steps (step/day)	0.53	-0.40*	-0.14	-0.36*
mean activity (count/day)	0.20	-0.25	-0.15	-0.16

BMI: body mass index

TEE: total energy expenditure per day

EEPA: energy expenditure originating from physical activity per day

^†^ : p<0.1, *: p<0.05, **: p<0.01, ***: p<0.001

With reference to television watching and video game playing, the results show that children who spent more time watching television or playing video games had lower levels of physical activity index, energy expenditure originating from physical activity, step, and mean activity, although significant differences were not found.

## DISCUSSION

Physical activity is considered to be an important component of healthy lifestyle. It is known that in developed countries the amount of habitual physical activity is decreasing enormously in child and adolescent periods. Accurate assessment of physical activity level in children is necessary to identify current levels of activity and to assess the effectiveness of intervention programs designed to increase physical activity.^19^^,^^21^ Physical activity can be assessed by subjective and objective methods that include questionnaire, direct observation, and mechanical devices.^22^^,^^23^ The physical activity questionnaire is probably the most commonly used method to assess physical activity in population studies because of its relatively low cost and ease of administration. Self-reported questionnaires evaluating the physical activity level of adolescents and adults have been widely used in epidemiologic surveys.^24^^,^^25^ However, according to Saris’s review in 1985, the commonly used measure of children’s self-reported questionnaires for physical activity cannot be used in children under the age of 10 years, because children of this age cannot give reliable information about their activity patterns.^26^ To study this premise further and in order to estimate data from the epidemiologic study, there is a need for valid and reliable quantitative measures of physical activity in younger children.^13^^,^^27^

The purpose of this study was to examine the validity of a self-administered physical activity questionnaire for schoolchildren. According to the children’s characteristics, we designed a simple questionnaire to evaluate children’s physical activity levels from different aspects, such as participation in sports club, intensity and frequency of physical activity, and preference for physical activity when compared to their peers.

Objective measures of physical activity are often included in a study to validate the data from questionnaires. It has been previously validated as an objective monitor of children’s physical activity in field and laboratory settings.^19^ Data indicate that in field settings, accelerometry can be used to assess the intensity of children’s activity.^22^^,^^28^ A widely used method to validate the assessment of physical activity is the use of an activity monitor such as Caltrac, Tritrac, or the CSA accelerometer.^2^^,^^28^^,^^29^ In the present study, we used two small instruments (the Lifecorder and the Actiwatch) as objective measures. Physical activity index is calculated from the Lifecorder indicators of total energy expenditure per day and BMR. From the results of multiple linear regression models, we found that physical activity index was significantly associated with children’s participation in sports club, intensity and frequency of physical activity, but not physical activity preference. Daily steps and energy expenditure originating from physical activity are calculated from physical activity, and were also be able to be sensitive indicators of evaluating physical activity level, although the effect of height and weight is not easy to control. Because the Actiwatch is designed to monitor an individual’s gross motor activity, activity data counts originating from the Actiwatch may also represent physical activity level to some degree. In our study, activity count correlated significantly with daily steps from the Lifecorder(r=0.45, p<0.001). Children with a “vigorous” or high frequency of physical activity, as well as those who liked physical activity very much, had a high mean activity count when compared to their peers who did not. The physical activity index may be the most valuable indicator for assessing children’s physical activity level, in contrast to the other variables originating from the Lifecorder and Actiwatch. Different objective parameters of physical activity measure different aspects of behavior, and it may be unreasonable to expect two distinct measures to correlate highly.^7^^,^^30^

Regarding the self-reported physical activity questionnaire, children who reported participation in sports club, with “vigorous” intensity or with a high frequency of physical activity, had a higher level of objective indicators (i.e. physical activity index, energy expenditure originating from physical activity, steps, and mean activity per day). In regard to physical activity preference, children who liked physical activity or playing outside very much had a relatively high level than those who did not like or disliked activity, although a significant difference was not found between “like” and “dislike” for some objective indicators, such as physical activity index. These findings suggest that intensity and frequency of physical activity may play an important role in evaluating children’s physical activity level. Children who spent more time watching television or playing video games had a lower level of physical activity originating from the two instruments, although a significant difference was not found.

It is known that total energy expenditure is related to subjects’ weight. However, even though a heavy child may have a high total energy expenditure per day, it is impossible to conclude that his physical activity level is high. In this study, therefore, we mainly used the energy expenditure originating from physical activity, steps, and physical activity index to evaluate children’s physical activity level, instead of the total energy expenditure per day. Findings from this study also show that children who reported vigorous physical activity intensity or a high frequency of physical activity had a low level of total energy expenditure per day, while the energy expenditure originating from physical activity and steps were significantly high. Therefore the modification of total energy expenditure per day by weight would not affect the trends of the results.

According to data from the Actiwatch, the average total activity score and the number of minutes of movement during sleep periods over seven consecutive days were not significantly different for the different physical activity levels reported by the children themselves. Therefore, the data presented in the results could be used to represent daily activity, to compare the different groups reported by the children.

In interpreting the results from this study, some limitations must be noted. First, the study was conducted from July through September in 2002. Because the Lifecorder monitor was not waterproof, subjects were asked to take off their monitor when they taking a shower or bath. This would underestimate the data from the Lifecorder. In fact, during the observation periods, each subject took a shower or bath for about 30 minutes every evening, as observed from the Lifecorder actigraphics. This small habitual behavior relating to children taking off their monitor would not have a great effect on the actual data collection. Second, only boys were observed in this study because of the limited number of instruments available and for measurement convenience. Nevertheless, the level of physical activity reported by children was positively related to objective data from the two instruments. Third, considering children’s physical activity patterns, as well as the characteristics of the accelerometer-based Lifecorder activity monitor used in this study, extrapolation of these results to some kinds of children’s specific physical activity types would be inadequate. This limitation of our study should be addressed in future studies. Last, the present study examined the validity of the self-reported physical activity questionnaire, but did not evaluate its test-retest reliability. Evaluation of the self-reported questionnaire’s reliability is also needed in future studies.

In summary, this study examined whether the self-administered physical activity questionnaire for schoolchildren could be validated by the Lifecorder and Actiwatch as criterion standards. The conclusion from this study is that self-reported physical activity levels are relatively in accordance with objective data from the two kinds of instruments. Consequently, the simple self-reported physical activity questionnaires of schoolchildren could be applied to a large population in an epidemiologic study.
